# Intraosseous clear cell mucoepidermoid carcinoma in the maxilla: A case report and review of literature

**DOI:** 10.1002/ccr3.4447

**Published:** 2021-07-23

**Authors:** Fumiya Harada, Yoshihiro Abiko, Ariuntsetseg Khurelchuluun, Koki Yoshida, Shigehiro Takeda, Eiji Nakayama, Tsuyoshi Shimo, Hiroki Nagayasu

**Affiliations:** ^1^ Department of Oral and Maxillofacial Surgery Division of Human Biology and Pathophysiology School of Dentistry Health Sciences University of Hokkaido Hokkaido Japan; ^2^ Department of Oral Medicine and Pathology Division of Human Biology and Pathophysiology School of Dentistry Health Sciences University of Hokkaido Hokkaido Japan; ^3^ Department of Reconstructive Surgery for Oral Maxillofacial Region Division of Human Biology and Pathophysiology School of Dentistry Health Sciences University of Hokkaido Hokkaido Japan; ^4^ Department of Oral Maxillofacial Radiology Division of Human Biology and Pathophysiology School of Dentistry Health Sciences University of Hokkaido Hokkaido Japan

**Keywords:** mucoepidermoid carcinoma, clear cell variant, intraosseous, maxilla

## Abstract

We reported an extremely rare case regarding intraosseous clear cell variant of mucoepidermoid carcinoma in maxilla.

## Graphical abstract

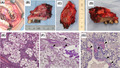

## 1 INTRODUCTION

Clear cells predominant mucoepidermoid carcinoma (cMEC) is a variant of mucoepidermoid carcinoma (MEC) which is barely seen. Primary intraosseous MECs are rare in the maxilla. Herein, we report for the first time, the case of a primary intraosseous cMEC in the maxilla.

Mucoepidermoid carcinoma (MEC) is composed of squamoid, mucin‐producing, and intermediate‐type cells. When clear cells predominate over the other cell types, it is called as a clear cell variant of MEC (cMEC).^1^ It is often difficult to distinguish cMEC from other clear cell‐containing salivary gland tumors such as clear cell carcinoma, epithelial‐myoepithelial carcinoma, and acinic cell carcinoma, and other tumors such as clear cell odontogenic carcinoma (CCOC) and metastatic renal cell carcinoma.^2^, ^3^ The MECs commonly occur in the parotid and palatal glands; only 4% of MECs have a primary intraosseous origin.^4^ The intraosseous MECs mainly arise in the mandible, and cases in the maxilla are extremely rare. To the best of our knowledge, cMEC arising in the maxilla in the form of an intraosseous tumor has not been reported thus far.

Herein, we reported a case of a 66‐year‐old male patient diagnosed with an intraosseous cMEC in the maxilla. In addition, a literature review of cases of intraosseous MEC and cMEC in the orofacial region is presented in this report.

## 2 CASE REPORT

A 66‐year‐old man was admitted to our clinic, Health Sciences University of Hokkaido hospital, in January 2017 due to pain in the gingiva on the left side of the maxilla. The pain had begun 4 to 5 years earlier. The patient had received root canal treatment of #27 in several dental clinics to treat the pain; however, the pain continued to persist. A panoramic X‐ray image was taken previously at a dental clinic showed an oval, multilocular, radiolucent lesion in the left maxilla. The patient was referred to our clinic for a detailed examination of the lesion.

His past medical history revealed the presence of hyperuricemia, Hepatitis C, and prostatic cancer. The prostatic cancer, diagnosed as adenocarcinoma, was excised in 2006 and had not recurred until 2015 when he stopped visiting the medical clinic. Subsequent follow‐ups were not performed, but the patient was asymptomatic so far.

**FIGURE 1:**
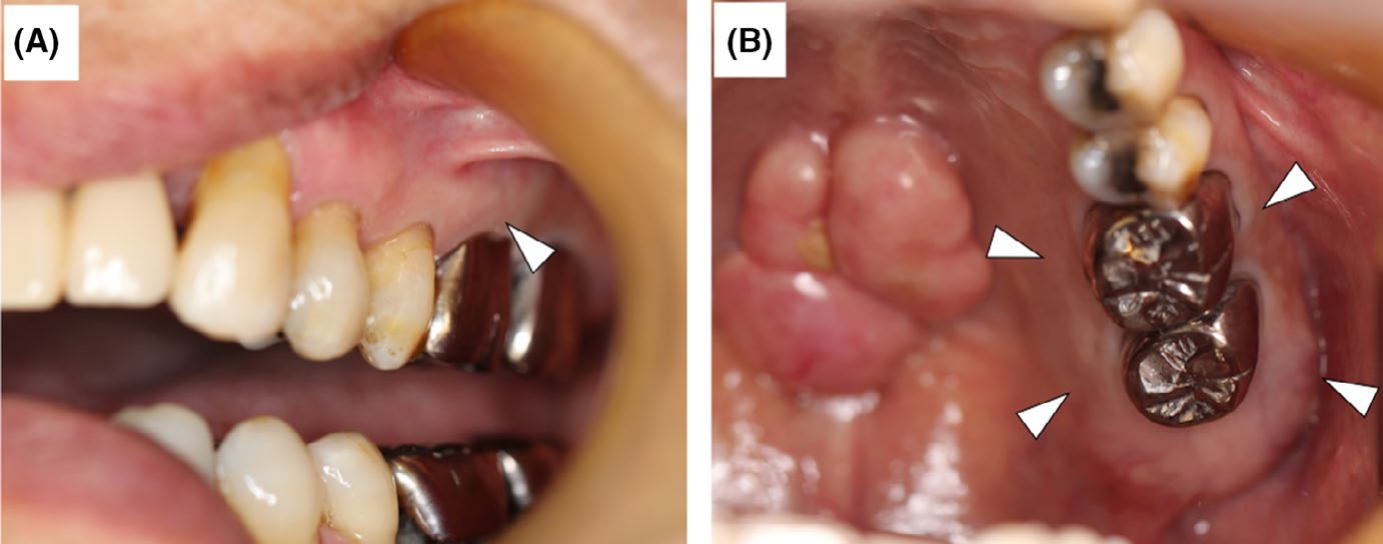
Intraoral appearance. A, Gingival mucosa was swollen in the left maxillary molar region. B, No swelling in the palatal mucosa. A palatal torus was found in the middle of the hard palate

He had a symmetrical facial appearance with no masticatory dysfunction or swelling of the lymphatic nodes. The gingiva in the left maxillary molar appeared smooth and healthy. However, swelling of the hard tissue was observed in the region with a parchment‐like appearance in the posterior region of #27. The patient felt pain on percussion in #26 and #27, but the teeth were not mobile (Figure 1A, B).

**FIGURE 2:**
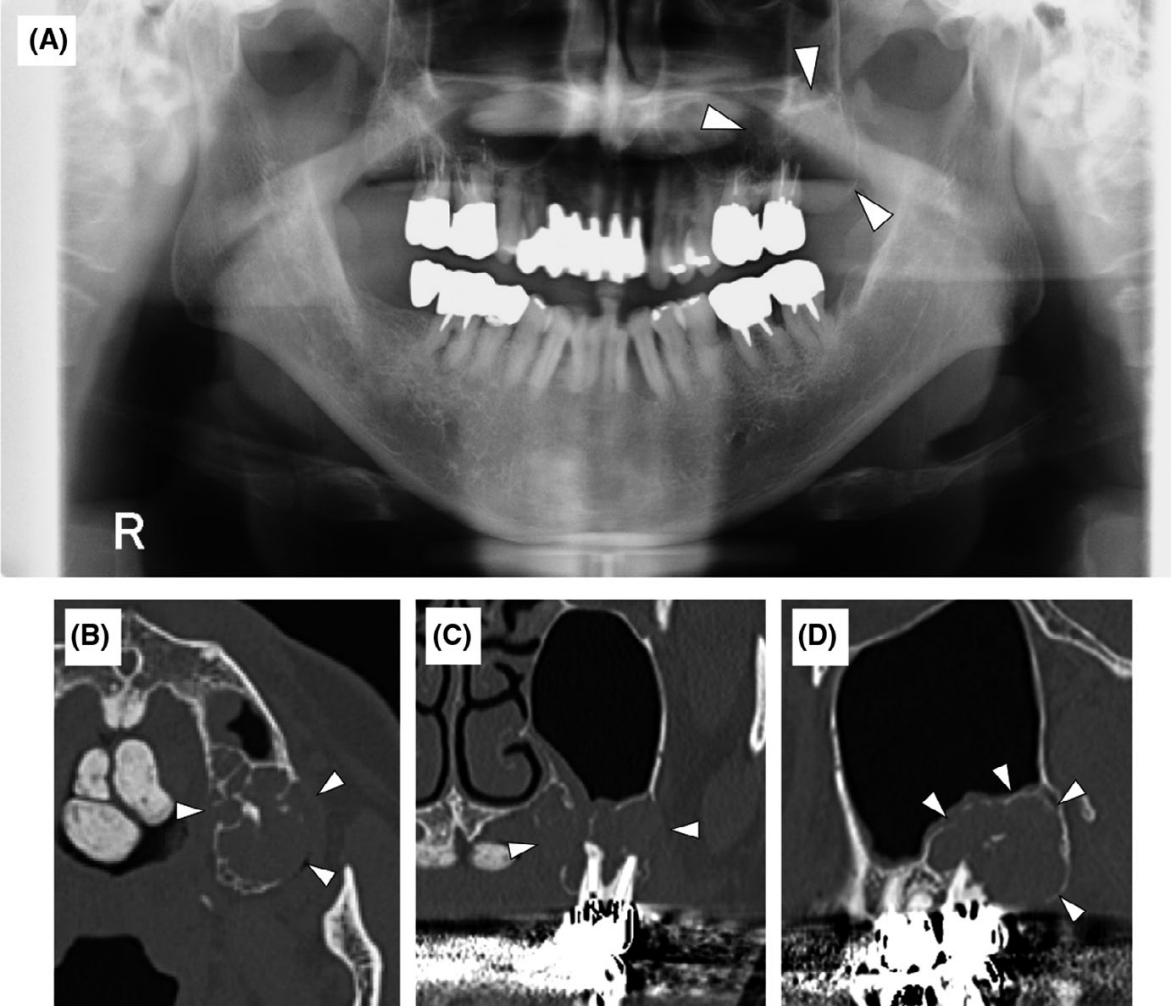
Panoramic X‐ray and Computed tomography (CT) image. A, Panoramic X‐ray showed a multilocular radiolucent lesion in the left maxillary posterior region (arrowhead). B, C, D, CT scan showed a multilocular lesion in the left maxillary posterior region. The alveolar bone appeared thin and partially discontinuous (arrowhead)

The X‐ray image showed an oval multilocular translucent area in the apical region of #27 (Figure 2A). Computed tomography (CT) revealed a well‐defined solid lesion in the left maxillary molar region. A bony, ridge‐like herd tissue was observed in the lesion, which appeared to apply pressure on the buccal, palatal, and sinus floor bone, and partially absorb the peripheral bone and #27 root. No obvious invasive findings in the sinus or gingiva were observed (Figure 2B, C, D).

**FIGURE 3:**
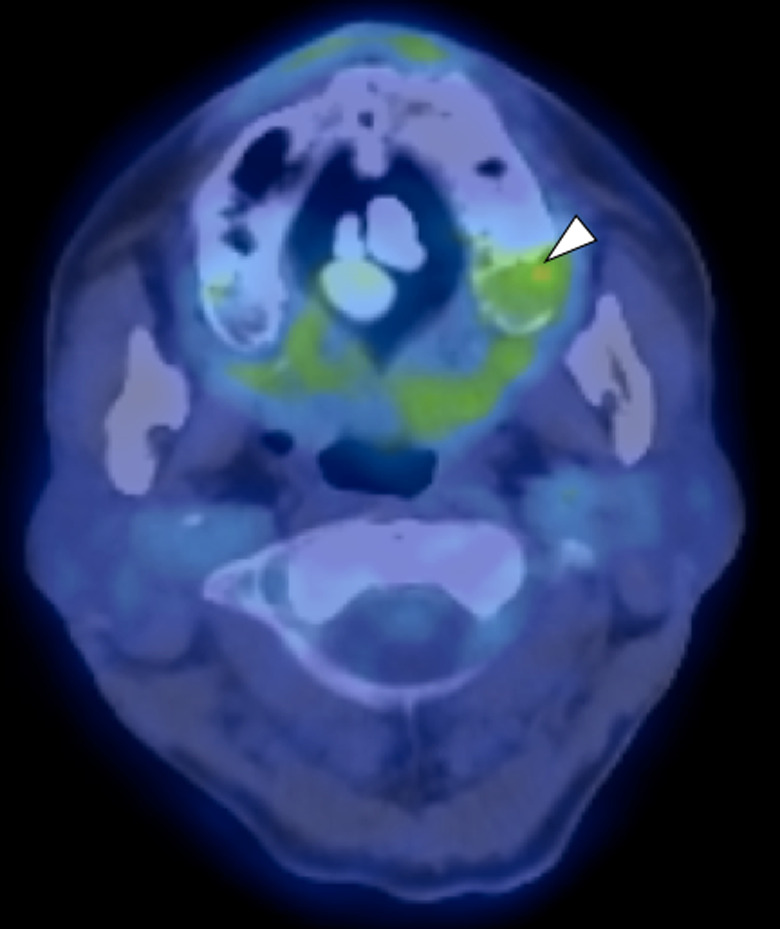
FDG‐PET image. The max‐SUV was 3.4 in the left maxillary lesion (arrowhead). No evidence of metastasis was found in the other organs

Fluorodeoxyglucose‐positron emission tomography (FDG‐PET) was systemically performed because bony metastasis of prostate cancer was suspected as a result of the bone invasion. FDG accumulation was observed in the left maxilla (Max‐standardized uptake value [SUV] = 3.4) and left middle lung field (Max‐SUV=6.7). No other abnormal accumulation was observed (Figure 3). The patient was referred to the Department of Pulmonary Medicine in our hospital and the lesion in the left lung was diagnosed as nontuberculous eosinophilia.

Although FDG‐PET implied that the lesion was a benign tumor, we suspected malignancy because of the finding of bone invasion on the X‐ray. Consequently, a biopsy was performed on the distal gingiva of #27.

## 3 DIFFERENTIAL DIAGNOSIS, INVESTIGATIONS, AND TREATMENT

**FIGURE 4:**
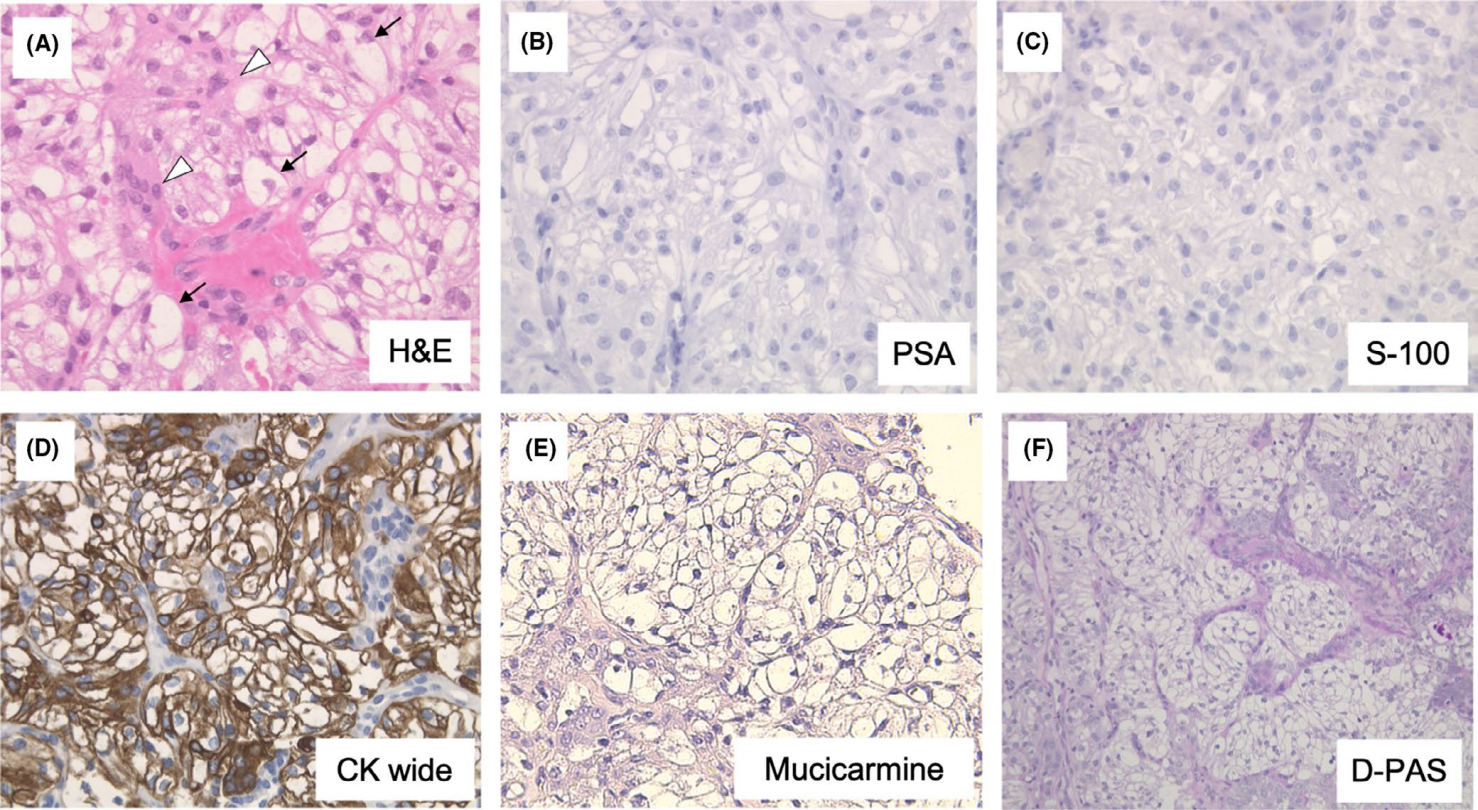
H&E, immuno‐histochemical and specific staining of biopsy. A, H&E staining showed clear cells (black arrow) and the epidermoid cells (white arrowhead) in the tumor cell nests. B‐D, Both PSA staining (B) and S‐100 staining (C) was negative. Cytokeratin (CK) wide staining was strongly positive for epidermoid cells (D). E&F: All clear cells were negative for mucicarmine (E) and diastase‐PAS staining (F)

The tumor tissue was mainly composed of clear cells. Epidermoid cells containing eosinophilic granules were observed in the tumor nests. However, no ductal structures were found in the nests. The stroma was narrow and composed of fibrous connective tissue with partial hyalinization. The cellular atypism was mild with few mitotic cells (Figure 4A). The possibility of metastasis of prostate gland carcinoma was ruled out due to negative staining for PSA (Figure 4B). Pleomorphic adenoma and epithelioma‐myoepithelioma were ruled out as a result of negative staining for S‐100 (Figure 4C). The presence of epidermoid cells was confirmed by positive staining for Cytokeratin (Figure 4D). Most parts of the tumor nests exhibited negative staining for mucicarmine or diastase‐PAS (Figure 4E, F). Odontogenic clear cell carcinoma was considered as the pathological diagnosis of the lesion at initial biopsy. The differential diagnoses based on clinical examination were central malignant tumor in the left maxilla (cT2N0M0 Stage II) and palatal torus.

**FIGURE 5:**
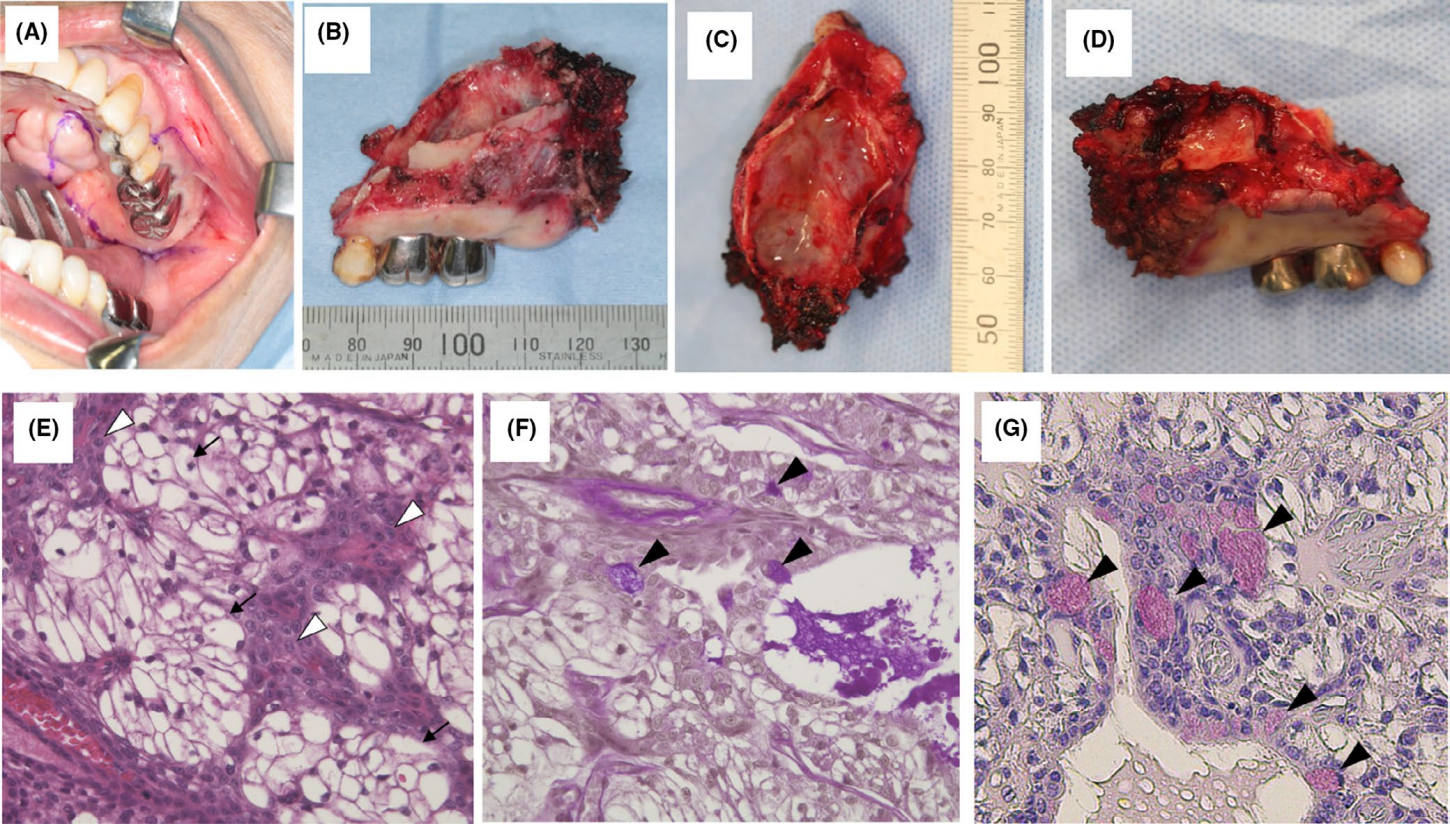
Incision line (A), resected specimen (B, C, D), and Hematoxylin and Eosin (E), Diastase‐PAS (F), and mucicarmine staining (G) of the excised specimens. A, The incision line was placed 10 mm away from the tumor margin. B, No tumor invasion into the buccal mucosa, but thinning of the buccal alveolar bone was observed. C, Smooth sinus mucosa and no tumor invasion into the sinus was observed. D, The palatal gingiva was smooth with no tumor invasion. E, Epidermoid cells (black arrow) and clear cells (white arrowhead) were observed in the tumor cell nests. F, Several clear cells were positive for diastase‐PAS (black arrowhead). G, Mucicarmine staining showed partially positive for mucin‐producing cells (black arrowhead)

Based on the diagnosis, partial osteotomy of the left maxilla and palatoplasty was performed under general anesthesia in March 2017. First, an incision was made on the mucosa of the palatal torus; the palatal gingiva was separated and the palatal torus was exposed. The torus was removed from the base. An incision line extending beyond the gingivobuccal fold was placed on the tumor. The safety margin was placed 10 mm away from the tumor, which was located at a distance from the palatal torus. After extraction #24, the maxillary bone at the medial part of the tooth was resected. On the upper side, the margin of the piriform aperture, the sinus floor, and the internal wall of the nasal cavity were resected; however, the infraorbital nerve was preserved. On the posterior side, a part of the pterygoid process was included. Incision line was designed as Figure 5A. Figure 5B, C, D show the incised tumor. The descending palatal artery was cauterized with an electric knife. The artificial dermis was placed over the wound followed by an Achromycin gauze dressing, which was then compressed with an oral appliance. A dental‐maxillary prosthesis was fabricated and placed; no harmful events were reported. Middle otitis was observed three months after the surgery, but it disappeared soon. It has been 15 months since the surgery; the surgical site has healed well and no recurrence or metastasis has been observed so far.

## 4 OUTCOME AND FOLLOW‐UP

In the excised tissue, the tumor cells had proliferated with the destruction of the bone at the floor of the sinus and partial exposure of the submucosal and mucosal layers of the oral epithelium. The tumor nests were mainly composed of clear cells, and epithelioid cells that were immune‐positive for p40 and p63 were observed around the nests. The cellular atypia was mild with a few mitotic cells. Some cystic and pseudo‐glandular structures were partially observed in the tumor nests. Microscopically, epidermoid cells and clear cells were observed in the tumor cell nests (Figure 5E). A part of the clear cells was stained with diastase‐PAS and mucicarmine staining indicating that mucin‐producing cells (Figure 5F, G). Metastasis of prostate cancer could be ruled out owing to the negative immunochemical staining for PSA. No tumor cells were found at the surgical margin. Based on these findings, a definitive diagnosis of a clear cell variant of mucoepidermoid carcinoma was made.

## 5 DISCUSSION

We here reported a case of comic arising as an intraosseous tumor in the maxilla. To our knowledge, this is the first case of an intraosseous cMEC in the maxilla. Only one case of intraosseous cMEC arising in the mandible has been reported in the past,^5^ whereas 29 cases of intraosseous MEC in the maxilla have been documented so far. The clinical data of intraosseous MEC in the maxilla are summarized in Table 1. The average age of onset of MEC is 36.4 ± 15.3 years, and the present case study was the oldest among intraosseous MEC.

**TABLE 1:** Reports of intraosseous MECs in the maxilla and cMEC in the oral and maxillofacial region

Age	Sex	Malignancy
Average =36.4 ± 15.5 (year‐old, N = 27)	M: F = 60.7:39.3 (%, N = 28)	G1: G2: G3 = 53.6:36.8:10.5 (%, N = 19)

Abbreviation: G; Grade (G1=Low, G2=Intermediate, G3=High).

The patient in this report was diagnosed with CCOC at initial biopsy because no mucin‐producing cells were detected. The presence of mucin‐producing cells in the excised tissue led to a definitive diagnosis of MEC. The diagnosis of cMEC was made according to the following diagnostic criteria ^1^:1) Solid proliferation of clear cells; 2) the presence of mucin‐producing cells, and 3) absence of clear cell carcinoma metastasis from the kidneys or the thyroid gland.

MECs are classified into 2 or 3 grades.^6^, ^7^, ^8^ In 2‐grade classification, low‐grade tumors are occupied by well‐differentiated mucin‐producing cells and epidermoid cells more than 50% of tumor nests, whereas in the high‐grade tumor are mainly occupied by intermediate‐ and poorly differentiated intermediate‐type cells with less than 10% of mucin‐producing cells. In 3‐grade classification, MEC has been divided into three grades based on pathological scores as follows: less than 20% cystic lesion (2 points); nerve invasion (2 points); necrotic nest (3 points); more than four mitoses at a magnification of 10× (3 points) and undifferentiation (4 points). The mild cellular atypia with few numbers of mitotic cells may indicate a low‐grade malignancy in the current case study. On the other hand, the nests consisted of less than 20% of cystic lesions, which corresponded to 2 points; therefore, it was categorized as a low‐grade malignancy according to the 3‐grade classification. However, in many cases, the histological grade does not correlate with the prognosis and low‐grade MECs may be treated aggressively.^8^ On the other hand, somatic gene translocation, t(11; 19), was strongly correlated with clear cell variant of MEC (100% occurrence), and the translocation was a factor of better overall survival ratio.^9^ Therefore, clear cell variant of MEC may be in better prognosis group than the others types of MEC.

The differential diagnoses of cMEC include CCOC, acinic cell carcinoma, clear cell ameloblastoma, clear cell oncocytoma, clear cell adenocarcinoma, epithelial‐myoepithelial carcinoma, myoepithelial carcinoma, and metastatic renal clear cell carcinoma.^2^, ^3^ The presence of clear cells has been a hallmark for the diagnosis of CCOC.^10^ In our case, an initial diagnosis of CCOC was reached at biopsy, because the clear cells were predominant and mucin‐producing cells were absent. The prognosis of MEC is different from that of CCOC. The 10‐year survival rates of low‐, intermediate‐, and high‐grade MEC are approximately 90%, 70%, and 25%, respectively.^11^ The death rate of CCOC is 15% with a median survival of 14 years.^12^ Although complete resection is essential for both MEC and CCOC, the definitive diagnosis should be made at initial biopsy. Irrespective of the absence of mucin‐producing cells, a possible diagnosis of cMEC should not be ruled out at initial biopsy.

Differential diagnoses of intraosseous MEC include glandular odontogenic cyst (GOC), central acinic cell carcinoma (CACC), and primary intraosseous squamous cell carcinoma (PIOSCC).^13^, ^14^, ^15^ GOT barely arises from central lesion of jaw bone, and microscopically appears MEC‐like tumor cell island in cyst wall.^13^ In addition, a case of transformation of GOT into MEC has been reported.^16^ Therefore, GOT should be carefully excluded from the diagnosis of MEC. Microscopic parameters for diagnosis of GOT are following; (1) eosinophilic cuboidal cells, (2) microcysts, (3) apocrine snouting, (4) clear (vacuolated) cells, (5) variable thickness, (6) tufting (papillary projections), (7) mucus cells, (8) epithelial spheres, (9) multiple compartments, and (10) cilia.^13^ The presence of 7 or more microscopic parameters was highly predictive of a diagnosis of GOT, while the presence of 5 or less microscopic parameters was highly predictive of a non‐GOC diagnosis.^13^ Only clear and mucus cells were obviously appeared in our case out of those 10 parameters.

Although about 90% of CACC arose in the parotid gland, several cases of ACC in central jaw bone have been reported.^14^, ^17^, ^18^, ^19^, ^20^, ^21^ CACC may be confused with well‐differentiated intraosseous MEC. ACC has a polymorphous appearance and is characterized as proliferation of acinic‐like cells containing serous components. ACC can be distinguished from the MEC with mucus cell dominant. SCC separated from oral mucosa in central jaw bone is called PIOSCC according to WHO classification.^22^, ^23^ Distribution of PIOSCC in mandible was 87.5% and in maxilla was 12.4%, respectively.^24^ PIOSCC can be easily distinguished from MEC with mucus components.

The possible origins of intraosseous salivary gland tumor are a neoplastic transformation of developmentally entrapped salivary glands or epithelia including an apical periodontal cyst, dentigerous cyst, mucosa of the maxillary sinus, and pluripotent reserve cells of excretory duct.^7^, ^14^, ^25^, ^26^ The patient in the current report had a history of root canal treatment in the maxillary second molar and did not show any obvious continuity between the tumor and the maxillary sinus. Thus, the tumor may have originated from an apical periodontal cyst or entrapped minor salivary glands.

In conclusion, this is the first report of a case of cMEC arising as an intraosseous tumor in the maxilla. A definitive diagnosis of cMEC should be made carefully because in some cases, only a few mucin‐producing cells may be present within the tumor.

## CONFLICT OF INTEREST

The authors have no conflicts of interest directly relevant to the content of this article.

## AUTHOR CONTRIBUTIONS

F. H., Y. A., and H. N. conceptualized; F. H., N. E., A. K., S. T., and H. N. prepared for the case presentation; F. H. and Y. A. wrote the original draft; F. H., Y. A., K. Y., E. N., T. S., and H. N. reviewed and edited the article;; F. H., E. N., and H. N. did the interpretation of radiograph, CT and PET image, F. H. visualized the article F. H., Y. A., K. Y., T. S., and H. N. supervised the work. All authors have read and agreed to the published version of the manuscript.

## ETHICAL STATEMENT

We explained to the patient about this case report and obtained his consent.

## Data Availability

The data that support the findings of this study are available from the corresponding author upon reasonable request.
